# Secretory Leucoprotease Inhibitor (SLPI) Promotes Survival during Acute *Pseudomonas aeruginosa* Infection by Suppression of Inflammation Rather Than Microbial Killing

**DOI:** 10.3390/biom12121728

**Published:** 2022-11-22

**Authors:** Megan Osbourn, Aoife M. Rodgers, Alice V. Dubois, Donna M. Small, Fiachra Humphries, Nezira Delagic, Paul N. Moynagh, Sinéad Weldon, Clifford C. Taggart, Rebecca J. Ingram

**Affiliations:** 1 Wellcome-Wolfson Institute for Experimental Medicine, Queen’s University Belfast, Belfast BT9 7BL, UK; 2 Department of Biology, The Kathleen Lonsdale Institute for Human Health Research, Maynooth University, W23 F2H6 Maynooth, Ireland; 3 The Patrick G Johnston Centre for Cancer Research, Queen’s University Belfast, Belfast BT9 7BL, UK

**Keywords:** secretory leucoprotease inhibitor, SLPI, inflammation, infection, Pseudomonas

## Abstract

Secretory leucoprotease inhibitor (SLPI) has multifaceted functions, including inhibition of protease activity, antimicrobial functions, and anti-inflammatory properties. In this study, we show that SLPI plays a role in controlling pulmonary *Pseudomonas aeruginosa* infection. Mice lacking SLPI were highly susceptible to *P. aeruginosa* infection, however there was no difference in bacterial burden. Utilising a model of *P. aeruginosa* LPS-induced lung inflammation, human recombinant SLPI (hrSLPI) administered intraperitoneally suppressed the recruitment of inflammatory cells in the bronchoalveolar lavage fluid (BALF) and resulted in reduced BALF and serum levels of inflammatory cytokines and chemokines. This anti-inflammatory effect of hrSLPI was similarly demonstrated in a systemic inflammation model induced by intraperitoneal injection of LPS from various bacteria or lipoteichoic acid, highlighting the broad anti-inflammatory properties of hrSLPI. Moreover, in bone-marrow-derived macrophages, hrSLPI reduced LPS-induced phosphorylation of p-IkB-α, p-IKK-α/β, p-P38, demonstrating that the anti-inflammatory effect of hrSLPI was due to the inhibition of the NFκB and MAPK pathways. In conclusion, administration of hrSLPI attenuates excessive inflammatory responses and is therefore, a promising strategy to target inflammatory diseases such as acute respiratory distress syndrome or sepsis and could potentially be used to augment antibiotic treatment.

## 1. Introduction

*Pseudomonas aeruginosa* is a Gram-negative opportunistic pathogen responsible for a range of infections, often with fatal outcome in immunocompromised or hospitalised patients [1]. It is a frequent cause of severe nosocomial pneumonia, which can result in acute respiratory distress syndrome (ARDS) and secondary sepsis [2]. During acute infection, *P. aeruginosa* induces robust inflammatory responses, which can result in pathogen clearance [3]. Paradoxically, dysregulated inflammation can result in tissue injury and life-threatening conditions including ARDS and sepsis. These acute inflammatory syndromes still lack therapeutic interventions to manage them, and as such, novel therapeutic strategies are warranted.

Secretory leucoprotease inhibitor (SLPI) is a non-glycosylated, 11.7 kDa monomeric protein and a member of the whey-acidic protein (WAP) family [4]. It is produced at mucosal surfaces, primarily by the epithelium of the upper respiratory tract [5], and by immune cells including macrophages, neutrophils and dendritic cells [6,7,8,9]. Its expression can be altered by a number of stimuli, notably by LPS, neutrophil elastase and various pro-and anti-inflammatory cytokines [7,10,11,12,13,14]. SLPI functions as a tissue protector, protecting against the deleterious consequences of excessive inflammation via its antiprotease activities, in addition to its antimicrobial and anti-inflammatory properties. SLPI inhibits elastase, cathepsin G, trypsin, chymotrypsin, chymase and tryptase, thereby counteracting the action of these proteases and curtailing the tissue damage that would otherwise ensue. Independent of its antiprotease activity, the broad-spectrum antibacterial, antifungal and antiviral properties of SLPI have also been reported [15,16,17,18]. SLPI has been found to limit the growth of *Escherichia coli*, *P. aeruginosa*, *Staphylococcus aureus*, *Aspergillus fumigatus* and *Candida albicans* [15,16,17,18,19,20,21].

The immunomodulatory activities of SLPI have been demonstrated both in vitro and in vivo [22]. In a model of LPS-induced endotoxin shock and sepsis induced by cecal ligation and puncture, SLPI deficient mice had increased mortality in comparison to that of wild-type mice. This may be in part explained by the increased production of IL-6 and increased NFκB activities by macrophages following LPS treatment [23]. Moreover, in a murine model of lung injury, SLPI administration resulted in reduced lung injury and prevented NFκB activation by inhibiting degradation of the NFκB inhibitor protein IκBβ [24,25]. SLPI blockade also resulted in intensification of lung injury and increased neutrophil accumulation [26]. In vitro, mechanistically it has been shown that SLPI may block binding of LPS to soluble CD14, and the subsequent movement of LPS from CD14 into cell membranes [14]. Cytosolic SLPI prevents LPS-induced NFκB activation by inhibiting degradation of IκBα and IκBβ [27], and attenuates TLR2 and TLR4 signalling [28]. Furthermore, in the nucleus, SLPI can compete with p65, preventing its interaction with the NFκB consensus region [5].

SLPI has long been recognised as a potential therapeutic candidate in chronic inflammatory lung diseases characterised by dysregulated protease activity, such as cystic fibrosis and chronic obstructive pulmonary disease [29,30,31,32]. To date, the potential of SLPI as a therapeutic for acute inflammation, and evaluation of SLPI’s anti-bacterial activity in vivo has been poorly investigated. As previously alluded to, in vitro SLPI has been purported to have antimicrobial properties against Gram-negative bacteria, including *P. aeruginosa*. Accordingly, within this study, we sought to establish if SLPI could ameliorate pulmonary *P. aeruginosa* infection and inflammation in vivo and establish the mechanism involved.

## 2. Materials and Methods

Experimental animals; C57BL/6 mice, originally purchased from Charles River Laboratories (Saffron Walden, UK), were bred in house. SLPI knockout mice (SLPI-KO) mice were a kind gift from Koji Atarashi [33] and subsequently bred in house. For all experiments, sex- and age- (12–16 wk old) matched mice were used. All animal work was conducted in accordance with the Animals Scientific Procedures Act (1986). The research was ethically reviewed by both the University Animal Welfare and Ethical Review Body (AWERB) and the Northern Ireland Dept of Health. The research was carried out under approved project licenses PPL2700 and PPL2807. 

In vivo LPS-induced Inflammation Models; Mice were instilled LPS (Sigma-Aldrich, Poole, UK) intratracheally under anaesthesia as previously described [34]. In short, mice were held upright on an intubation platform and administered 20 µg of *P. aeruginosa* LPS or PBS (in 50 µL) using a MicroSprayer aerosoliser attached to a high-pressure syringe (Penn-Century, Philadelphia, PA, USA). This was positioned through the vocal cords using a mouse laryngoscope (Harvard Apparatus, Cambridge, UK) [35]. Ten minutes post LPS administration, mice received either PBS, 100 µg human recombinant (hr) SLPI (Amgen, Thousand Oaks, CA, USA) or 100 µg ovalbumin (Sigma-Aldrich) delivered intraperitoneally (in 100 µL). For the systemic inflammation model, mice received 250 µg of *P. aeruginosa* LPS, *K. pneumoniae* LPS, *E. coli* LPS or *S. aureus* LTA (in 100 µL) delivered intraperitoneally. As before, ten minutes post LPS administration, PBS or 100 µg hrSLPI was delivered intraperitoneally (in 100 µL). In all cases, mice were sacrificed 6 h or 24 h later and bronchoalveolar lavage fluid (BALF) or peritoneal lavage fluid was collected. 

In Vivo Bacterial Infection; In vivo infections were carried out as previously described [36]. In short, a log phase culture of *P. aeruginosa* (Q502) was washed and resuspended in endotoxin-free PBS at an OD (600 nm) of 0.5. Mice were anesthetised and intranasally inoculated with 20 µL of *P. aeruginosa* or saline control. Mice were subsequently administered hrSLPI as described above and sacrificed 24 h post infection. Lung homogenate was serially diluted and plated on cetrimide agar (Sigma-Aldrich) and incubated overnight at 37 °C for quantification of colony forming units (CFUs). 

Lung Histology; Histology was performed as previously described. Whole lungs were fixed in 10% formalin (Sigma-Aldrich, UK) for 48 h, embedded and sectioned for staining with Harris haematoxylin (Thermo Scientific, Horsham, UK) and eosin (Leica, Milton Keynes, UK). Images were taken using a Leica DM5500B microscope (Leica, UK) and analysed with Leica AL software3.

Flow Cytometry; Cells were centrifuged at 300× *g* for 10 min at 4 °C and 10^6^ cells were stained for flow cytometry. Briefly, Fc receptors were blocked for 15 min with anti-CD16/CD32 (eBioscience, San Diego, CA, USA), the cells were then washed and stained with antibodies against GR1-PE (clone GR-1), CD11b-APC (clone M1/70), F4/80-PE-Cy7 (clone BM8), (eBioscience) and CD3-APC-Cy7 (clone 145-ZCII) (Biolegend, San Diego, CA, USA). Cells were washed and resuspended in PBS for acquisition on a FACSCanto II cytometer (BD Biosciences). Data was analysed using FlowJo software v3.0 (Tree Star). Neutrophils were defined as GR-1+ CD11b+, macrophages as GR1- F4/80+ CD11b+ and T cells as CD3+ cells. The absolute number of cells were calculated using the percentages and total cell counts performed on the original sample.

Bone Marrow Derived Macrophages; Isolation of bone marrow derived macrophages (BMDMs) was performed as previously described [37]. The tibias and femurs of wild-type and SLPI-KO mice were flushed with fresh RPMI-1640 plus GlutaMAX-I medium using a 27^1/4^ gauge needle. Cells were plated in medium supplemented with 10% (*v*/*v*) conditioned medium of L929 mouse fibroblasts. Cells were maintained for 6 days at 37 °C in a humidified atmosphere of 5% CO_2_. The obtained BMDMs were cultured in 12-well plates (1 × 10^6^ cells per mL; 1 mL), pre-treated with 25 µg/mL hrSLPI for 30 min, and then stimulated with 100 ng/mL of LPS from *P. aeruginosa* for 30 min or 1 h.

Enzyme-Linked Immunosorbent Assay (ELISA); Levels of IL-6, KC and MCP-1 in BAL or peritoneal lavage fluid were quantified according to manufacturer’s instructions (R&D Systems (Minneapolis, MI, USA) and eBioscience).

Real-time PCR analysis; Perfused whole lungs were harvested and snap frozen in liquid nitrogen. Lungs were homogenised in 1 mL of Trizol reagent according to manufacturer’s instructions. cDNA was generated from 1 µg RNA using cDNA Synthesis kit (Bio-rad, UK). Real-time PCR analysis was performed with GoScript TM Reverse Transcription System according to manufactures instructions. The housekeeping gene GAPDH was used for all experiments. Mouse IL-1β, forward, CAACCAACAAGTGATATTCTCCATG and reverse GATCCACACTCTCCAGCTGCA; Mouse IL-6 forward GTTCCTCTCTGCAAGAGACTTCC and reverse GTATCCTCTGTGAAGTCTCCTCTCC; Mouse TNFα forward, CCCTCACACTCAGATCATCTTCT, and reverse GCTACGACGTGGGCTACAG; IFNγ forward TGAGTATTGCCAAGTTTGAGGTCA and reverse CGGCAACAGCTGGTGGA.

Western blotting; For whole cell lysate analysis, cells were lysed in NP-40 lysis buffer (50 mM Tris-HCl, pH 7.4, containing 150 mM NaCl, 1% (*w*/*v*) IgePal, 50 mM NaF, 1 mM Na_3_VO_4_, 1 mM dithiothreitol, 1 mM phenylmethylsulfonyl fluoride and complete protease inhibitor mixture (Roche)). Samples were resolved by SDS-PAGE, transferred to nitrocellulose membranes, and subsequently analysed by immunoblot with the indicated antibodies (Cell Signalling; anti-pIkBa (9246), anti-pIkk (2697) and anti-p-p38 (4511), and anti β-actin antibody (A5316) (Sigma-Aldrich). Immunoreactivity was visualized by the Odyssey Imaging System (LICOR Biosciences, Cambridge, UK), or enhanced chemiluminescence.

Statistical analysis; All data were analysed using GraphPad Prism (GraphPad Software, San Diego, CA, USA). The normality of the samples’ distribution was assessed by the D’Agostino and Pearson omnibus normality test or by the Kolmogorov–Smirnov test. The normally distributed data sets were compared using a one-way ANOVA with Bonferroni post-test or an unpaired t-test, while non-normally distributed data was compared using the non-parametric Kruskall-Wallis test with Dunns post-test, or the Mann–Whitney test. All data points are represented on the graphs along with the mean (for normally distributed data sets) or the median (for non-normally distributed data sets) of the data. Significant differences are represented by * *p* < 0.05, ** *p* < 0.01 or *** *p* < 0.001; ns (non-significant) corresponds to *p* > 0.05.

## 3. Results

### 3.1. SLPI-Deficient Mice Are Highly Susceptible to Pulmonary Pseudomonas Aeruginosa Infection

**Figure 1:**
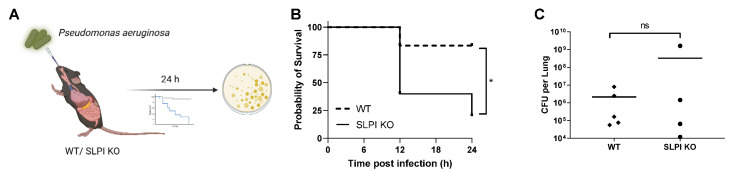
SLPI KO mice are more susceptible to PA lung infection than that of wild-type mice. WT or SLPI KO mice were infected intranasally with *P. aeruginosa* (2 × 10^7^) and survival was monitored, or CFU were collected 24 h post infection (**A**). There was a significant decrease (*p* = 0.04) in survival in SLPI KO mice (*n* = 6 per group) (**B**). There was no difference in the CFU titres observed in lung homogenate 24 h (**C**). Symbols are representative of individual mice with bars representing mean CFU numbers, *n* = 4–5 mice, * (*p* < 0.05), ns (non-significant) corresponds to *p* > 0.05.

To assess the role of SLPI during *P. aeruginosa* infection, wild-type and SLPI deficient (SLPI-KO) mice were intranasally challenged with *P. aeruginosa* and their survival was monitored (Figure 1A). SLPI KO mice had a higher susceptibility to acute *P. aeruginosa* infection in comparison to their wild-type counterparts. At 24 h post infection, 83% of wild-type mice had survived the infection, whereas approximately 30% of SLPI-KO mice survived (Figure 1B). Subsequent enumeration of CFU titres in the lungs of mice post infection, highlighted that while wild-type mice had increased survival, there were no significant differences in the lung bacterial burden between wild-type and SLPI-KO mice (Figure 1C). Moreover, there was no significant difference in the bacterial burden in the spleens or liver (data not shown). These results indicate that SLPI-KO mice are highly susceptible to *P. aeruginosa* acute lung infection and therefore, SLPI plays a key role in the controlling host defence against *P. aeruginosa* infection in the lung.

### 3.2. Endogenous SLPI Is Involved in Controlling LPS-Induced Lung Inflammation

**Figure 2:**
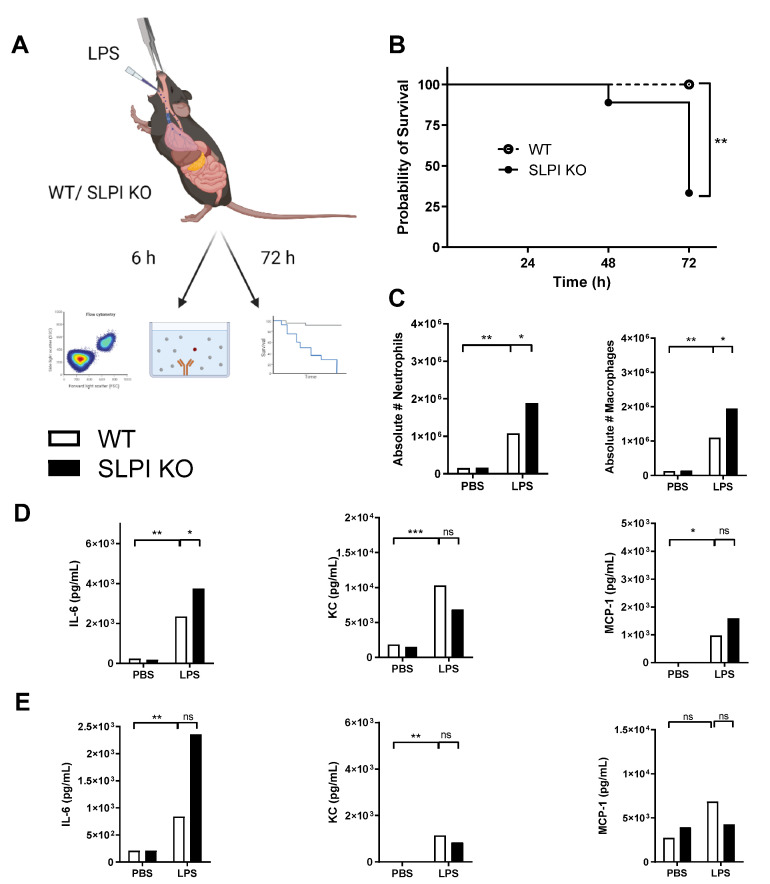
SLPI is involved in controlling local LPS-induced lung inflammation. C57bl6 WT or SLPI KO mice were instilled with PBS or LPS (20 µg) intratracheally (**A**). The survival in C57bl6 WT (white dots) or SLPI KO (black dots) was monitored over 72 h (*n* = 9 group) (**B**) and a significant difference was observed (*p* = 0.004). The BAL was analysed for neutrophils and macrophages (**C**) 6 h post-instillation. The levels of IL-6, KC and MCP-1 were quantified by ELISA in BALF (**D**) and in the serum (**E**). * *p* < 0.05, ** *p* < 0.01 or *** *p* < 0.001; ns (non-significant) corresponds to *p* > 0.05.

Given that SLPI KO mice were highly susceptible to *P. aeruginosa* lung infection, but had no differences in bacterial CFUs, a *P. aeruginosa* LPS-induced lung inflammation model was utilised to further investigate this finding (Figure 2A). Paralleling results from *P. aeruginosa* infection studies, survival of SLPI KO mice was significantly lower than that of wild-type mice in response to LPS-induced lung inflammation, therefore suggestive of a decreased ability of these mice to resolve inflammation (Figure 2B). In the acute inflammatory phase, 6 h post-instillation, LPS induced an increase in the recruitment of immune cells in BALF, notably neutrophils and macrophages. SLPI KO mice had significantly higher numbers of neutrophils and macrophages at this time-point (Figure 2C). The concentration of the pro-inflammatory cytokine IL-6, the neutrophil chemoattractant KC and the monocyte chemokine MCP-1 were also increased upon LPS stimulation, both locally (Figure 2D) and systematically (Figure 2E). There were no significant differences in the concentrations of KC or MCP-1 between wild-type and SLPI KO mice. At 6 h post infection, SLPI KO mice did, however, have significantly higher BAL fluid levels of IL-6 than that of wild type mice (Figure 2D). Taken together, this data demonstrates that endogenous SLPI is involved in the recruitment of inflammatory cells and protects against LPS-induced lung inflammation.

### 3.3. Administration of hrSLPI Decreases LPS-Induced Lung Inflammation

**Figure 3:**
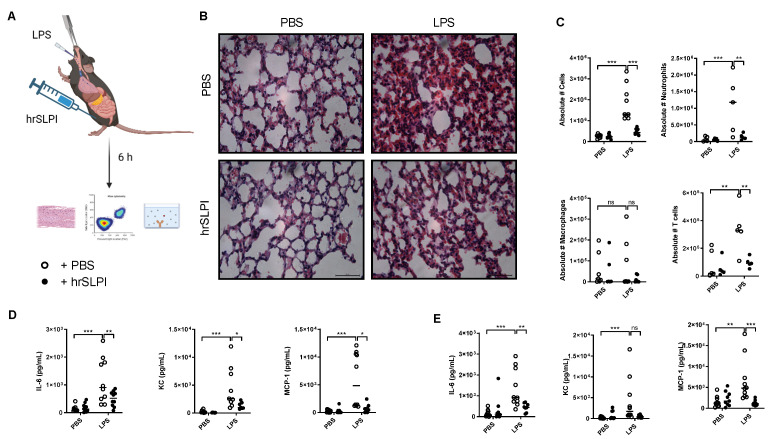
Administration of hrSLPI decreases LPS-induced lung inflammation. C57bl6 mice were instilled with PBS or 20 µg of LPS intratracheally and injected intraperitoneally with PBS or 100 µg hrSLPI (**A**). After 6 h, lungs were fixed and stained with hematoxylin & eosin (**B**) (bar = 50 µm), the total cells, neutrophils, macrophages and T cells were quantified within BAL (**C**) and the levels of IL-6, KC and MCP-1 were determined by ELISA in BALF (**D**) and serum (**E**) in the PBS (white dot) and hrSLPI (black dot) treated mice. * *p* < 0.05, ** *p* < 0.01 or *** *p* < 0.001; ns (non-significant) corresponds to *p* > 0.05.

**Figure 4:**
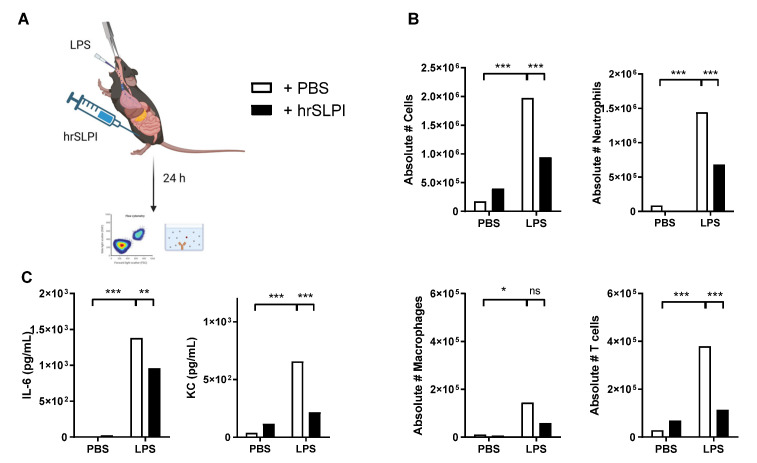
LPS-induced inflammation is still reduced 24 h after administration of hrSLPI. C57bl6 mice were instilled with PBS or 20 µg of LPS intratracheally and injected intraperitoneally with PBS or 100 µg hrSLPI (**A**). After 24 h, the total cells, neutrophils, macrophages and T cells were quantified in the BAL (**B**) and the levels of IL-6 and KC were determined by ELISA in the BALF (**C**) in PBS (white dots) or hrSLPI (black dots) treated mice. * *p* < 0.05, ** *p* < 0.01 or *** *p* < 0.001; ns (non-significant) corresponds to *p* > 0.05.

As endogenous SLPI could protect against both *P. aeruginosa* and LPS-induced lung inflammation and subsequent mortality, it was postulated that administration of recombinant SLPI would therefore be a possible strategy to reduce acutely driven lung inflammation. Accordingly, following induction of LPS-induced lung inflammation, human recombinant SLPI (hrSLPI) was delivered via intraperitoneal route (Figure 3A). Dose response experiments confirmed that the administration of 100 µg of hrSLPI significantly decreased the recruitment of immune cells to the lungs, while 20 µg and 50 µg had no significant effect (Supplementary Figure S1A). By ELISA, we confirmed that the hrSLPI injected in the peritoneum was efficiently absorbed in the blood and reached the lungs (Supplementary Figure S1B,C). Administration of hrSLPI resulted in decreased LPS-induced infiltration of cells as observed in histological samples of whole lung tissue from mice that received hrSLPI, in comparison to control mice (Figure 3B). The recruitment of neutrophils and T cells were both significantly decreased by hrSLPI (Figure 3C). Inflammatory markers such as IL-6, TNFα, IL-1β and IFNγ were all reduced at the mRNA level within whole lung tissue of treated mice (Supplementary Figure S2B). Using a cytokine array, we observed that hrSLPI administration reduced the concentration of a plethora of inflammatory cytokines and chemokines (Supplementary Figure S2A). Key cytokines were quantified by ELISA, this showed significant reductions in IL-6, KC and MCP-1 (Figure 3D). Serum IL-6 and MCP-1 were also significantly decreased following administration of hrSLPI (Figure 3E). The effect of hrSLPI on neutrophil and T cell recruitment, as well as on the concentration of IL-6 and KC, in the lung was maintained 24 h post hrSLPI administration (Figure 4A–C). We confirmed the anti-inflammatory effects of hrSLPI were not due to a response to a non-mouse protein, since injection of another foreign protein, ovalbumin, neither reduced the concentration of IL-6 and KC in the BALF or serum, nor the recruitment of immune cells (Supplementary Figure S3).

### 3.4. Administration of hrSLPI Decreases LPS-Induced Systemic Inflammation

**Figure 5:**
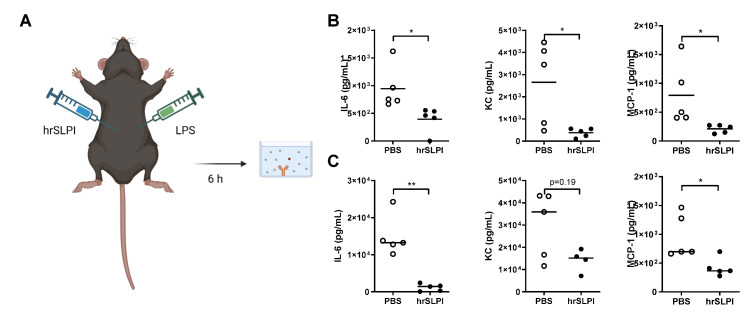
Administration of hrSLPI decreases LPS-induced systemic inflammation. C57bl6 mice were injected with 250 µg of LPS from *E. coli* intraperitoneally with concurrent administration of 100 µg hrSLPI or PBS control (**A**). After 6 h, the levels of IL-6, KC and MCP-1 were determined by ELISA in the peritoneal lavage (**B**) and in the serum (**C**) of hrSLPI (black dots) or PBS (white dots) treated mice (*n* = 5 per group). * *p* < 0.05 or ** *p* < 0.01.

In addition to lung inflammation, uncontrolled acute systemic inflammation can be life-threatening. Utilising a model of LPS-induced systemic inflammation by intraperitoneally administered LPS from *E. coli* (Figure 5A)*,* which is a more relevant systemic model for peritoneal infection than LPS from *P. aeruginosa*, the ability of hrSLPI to dampen systemic inflammation was investigated. Concurrent with previous results, administration of hrSLPI suppressed systemic inflammation, significantly reducing LPS-induced production of IL-6, KC and MCP-1 in the peritoneal lavage (Figure 5B). In the serum, the concentration of IL-6 and MCP-1 was significantly decreased in the presence of hrSLPI (Figure 5C). These results suggest that hrSLPI is capable of suppressing LPS-induced systemic inflammation. Importantly, this effect was not specific to LPS from *E. coli*, as a similar effect was also observed with LPS from other Gram-negative bacteria; *P. aeruginosa* (Supplementary Figure S4A) and *K. pneumoniae* (Supplementary Figure S4B), as well as with the Gram-positive pathogen-associated molecular pattern, lipoteichoic acid (LTA) of *S. aureus* (Supplementary Figure S4C). In all cases, the local production of IL-6 was significantly decreased by hrSLPI (Supplementary Figure S4A–C) and in the serum IL-6 was also significantly reduced by hrSLPI in the case of *K. pneumoniae* LPS and *S. aureus* LTA (Supplementary Figure S4B,C). Importantly, this demonstrates that the anti-inflammatory effects of hrSLPI are not specific of the PAMP inducing inflammation and that SLPI has broad-spectrum anti-inflammatory properties in vivo.

### 3.5. hrSLPI Interferes with the NFκB and MAPK Pathways

**Figure 6:**
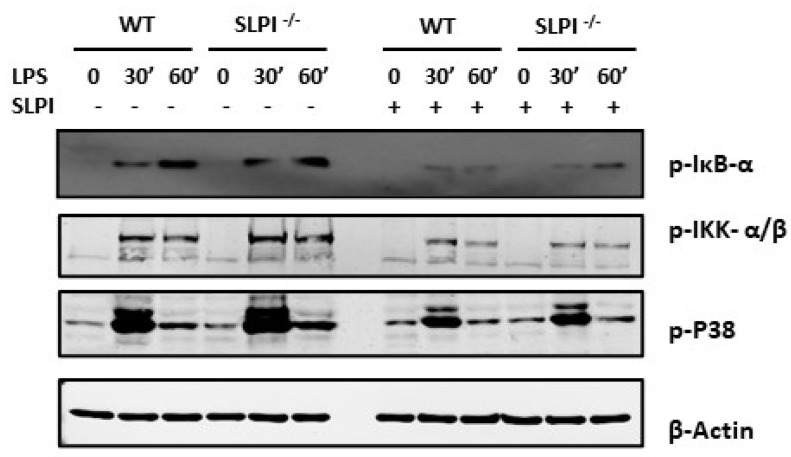
hrSLPI interferes with the NFκB and MAPK pathways. Bone-marrow-derived macrophages from WT or SLPI KO mice were stimulated with 100 ng/mL LPS for 0, 30 min or 60 min, with or without pre-treatment with 25 µg/mL hrSLPI for 30 min. The cell lysates were collected and analysed by Western blot for p-IkB-α, p-IKK-α/β, p-P38 and β-Actin.

To determine if the anti-inflammatory properties of hrSLPI were due to an effect on NFκB and MAPK pathways, we incubated bone-marrow-derived macrophages (BMDMs) from WT and SLPI deficient mice with LPS in the presence or absence of hrSLPI and performed Western blots analysis for key markers of these pathways. As expected, LPS treatment induced activation of NFκB and MAPK pathways, as measured by phosphorylation of IκB-α, IKK-α/β and the p38 MAP Kinase at both 30- and 60 min post stimulation (Figure 6). This effect was more pronounced in BMDMs derived from SLPI deficient mice, compared to that of WT mice (Figure 6). Furthermore, for both genotypes, pre-treatment of BMDM with hrSLPI reduced the phosphorylation of IκB-α, IKK-α/β and P38, demonstrating that SLPI interferes with the activation of both the NFκB and MAPK pathways (Figure 6).

## 4. Discussion

In the present study, we demonstrated that endogenous SLPI played a role in host defence against *P. aeruginosa infection*. SLPI KO mice were highly susceptible to *P. aeruginosa infection,* exhibiting increased mortality. Despite this, there were no significant differences in the bacterial burden in SLPI KO mice, indicating that SLPI does not play a role in direct bacterial killing. In vitro, SLPI has previously been purported to have antimicrobial properties against a number of bacteria including dermatological *S. aureus* and *P. aeruginosa* isolates [15]. In contrast, we have previously demonstrated that SLPI had no antimicrobial activity against clinical isolates of *P. aeruginosa* [38] and could not detect any anti-microbial effect of hrSLPI [36]. These contradictory reports may be due to differences in antimicrobial resistance between strains utilised within the two studies. To our knowledge, there have been no previous reports on the role of endogenous SLPI during *P. aeruginosa* infection in vivo. 

Having demonstrated the fundamental role of endogenous SLPI during *P. aeruginosa* infection, we sought to investigate if the decreased survival was due to suppression of inflammation. Using a model of *P. aeruginosa* LPS-induced lung inflammation, we demonstrate that endogenous SLPI was involved in regulating the level of IL-6 and the recruitment of neutrophils and macrophages to the lung. These results suggest that endogenous SLPI is involved in controlling the inflammatory response to protect the host. The expression of SLPI has already been shown to be increased by several pro-inflammatory stimuli, such as LPS [7], TNFα, IL-1β, [39], and neutrophil elastase [40] and elevated SLPI concentrations have been detected in various inflammatory diseases. High levels of SLPI can, for example, be detected in the serum of patients with sepsis [41], the BALF of patients with ARDS, or at risk of developing ARDS [42], as well as the BALF of patients with COPD [43,44]. Interestingly, COPD patients who suffer frequent exacerbations have reduced levels of SLPI in comparison to those with stable disease [45], suggesting that a lack of SLPI is detrimental in controlling inflammation.

The effect of endogenous SLPI in regulating inflammation indicates that the administration of exogenous SLPI may be helpful in reducing acutely driven lung inflammation. We used human recombinant SLPI (hrSLPI] to be able to discriminate it from the mouse’s endogenous SLPI and confirm its distribution to the blood and lung. Human recombinant SLPI is 58% homologous to mouse SLPI at the amino acid level, 80% at the peptide level, has only one variant residue in the inhibitory loop [46], and is able to inhibit mouse neutrophil elastase [47], which validates its use in a mouse model. Intraperitoneal administration of hrSLPI resulted in hrSLPI efficiently being transferred to the blood and the lung.

The administration of hrSLPI efficiently inhibited LPS-induced lung inflammation, as demonstrated by the number of neutrophils and T cells, as well as the concentration of the proinflammatory cytokine IL-6, the neutrophil chemokine KC and the monocyte chemokine MCP-1, which were at similar levels to those found in mice instilled with PBS alone. Additionally, using a cytokine array, we showed that the effect of hrSLPI was not limited to those three cytokines, greater than 30 inflammatory markers were decreased by hrSLPI. Reduced inflammation by hrSLPI was still observed 24 h post-LPS instillation, suggesting a prolonged effect of hrSLPI. Moreover, similar results were obtained in a systemic inflammation model, whereby hrSLPI administration reduced immune cell infiltration and inflammatory cytokine production in the peritoneum and serum following LPS challenge. 

The anti-inflammatory effects of hrSLPI were not PAMP-specific, as hrSLPI decreased inflammation induced by *E. coli* LPS, *P. aeruginosa* LPS, *K. pneumoniae* LPS and by the LTA from *S. aureus*. The activity of SLPI against LTA-triggered inflammation has previously been demonstrated in vitro [28]. Thus, this provides additional in vivo supportive evidence to indicate that SLPI is able to reduce LPS and LTA responses in macrophages [14,28]. Importantly, this highlights that hrSLPI could be used to decrease the inflammation induced by a variety of pathogens or stimuli.

Benefits of SLPI administration or overexpression have previously been suggested for various chronic inflammation models including asthma [48], emphysema [31], and arthritis [49] and more recently, colitis [50]. A clinical trial involving the administration of aerosolised hrSLPI to cystic fibrosis patients decreased IL-8 levels and elastase activity in BAL fluid, highlighting its potential in the treatment of human chronic lung disease [29]. To our knowledge, the therapeutic potential of SLPI to modulate acutely driven inflammation has been poorly investigated. 

Several mechanisms of action of SLPI have been described over the years; SLPI can bind LPS [14], preventing its interaction with TLR-4 [14], prevent the degradation of IκB [27], or preclude the binding of the p65 subunit of NF NFκB B to bind to the promotor of pro-inflammatory genes [5]. We observed that the administration of hrSLPI decreased the production of inflammatory cytokines both at the transcriptional and protein levels. Using bone-marrow derived macrophages (BMDMs), we confirmed the ability of hrSLPI to block the activation of the NFκB pathway. Additionally, we also demonstrated that hrSLPI could interfere with the MAPK pathway by showing that SLPI KO BMDMs displayed increased levels of phosphorylated p38 MAPK and that pre-treatment with hrSLPI decreased LPS-induced phosphorylation of p38. This ability of hrSLPI to inhibit the two main pro-inflammatory transduction pathways, the NFκB and the MAPK pathways, is reflected by our demonstration that hrSLPI reduces the production of more than 30 inflammatory markers, is efficient against inflammation triggered by multiple pathogen-associated molecular patterns and in both systemic and respiratory settings. The broad and non-specific anti-inflammatory power of hrSLPI that we demonstrated here makes SLPI and its derivatives attractive therapeutics to rapidly target acute inflammation.

## 5. Conclusions

In conclusion, SLPI plays a key role in controlling *P. aeruginosa* infection through suppression of inflammation rather than microbial killing. Administration of hrSLPI was able to reduce acutely driven inflammation in both the lung and systemically. As such, SLPI could be considered as a therapeutic for either sterile inflammatory conditions, or as a combined therapy in infected patients.

## Data Availability

Available from corresponding author upon reasonable request.

## Supplementary Materials

The following supporting information can be downloaded at: https://www.mdpi.com/article/10.3390/biom12121728/s1, Figure S1: hrSLPI administered intraperitoneally reaches the lung and decrease the lung cellular infiltrate; Figure S2: Administration of hrSLPI decreases the LPS-induced expression of numerous inflammatory cytokines; Figure S3: The hrSLPI anti-inflammatory effect is not due to the simple administration of a protein; Figure S4: hrSLPI anti-inflammatory properties are not specific of E. coli LPS.
