# Supplementary material for: Secretory Leucoprotease Inhibitor (SLPI) Promotes Survival during Acute Pseudomonas aeruginosa Infection by Suppression of Inflammation Rather Than Microbial Killing
Source: Biomolecules. 2022 Nov 22;12(12):1728. doi: 10.3390/biom12121728 (PMC9776001; doi:10.3390/biom12121728)

biomolecules

Figure S1. hrSLPI administered intraperitoneally reaches the lung and decrease the lung cellular infiltrate. C57bl6 mice were instilled with PBS or LPS (20 µg) intratracheally and treated with PBS (white dots), 20 µg (light grey dots), 50 µg (dark grey dots) or 100 µg (black dots) of hrSLPI intraperitoneally. The total cells were counted in the BAL 6 h post-instillation (**A**) and hrSLPI was quantified by ELISA in the serum (**B**) and the BAL (**C**) of the animals treated with 100 µg of hrSLPI. * ($p < 0.05$), ** ($p < 0.01$) or *** ($p < 0.001$); ns (non-significant) corresponds to $p > 0.05$.

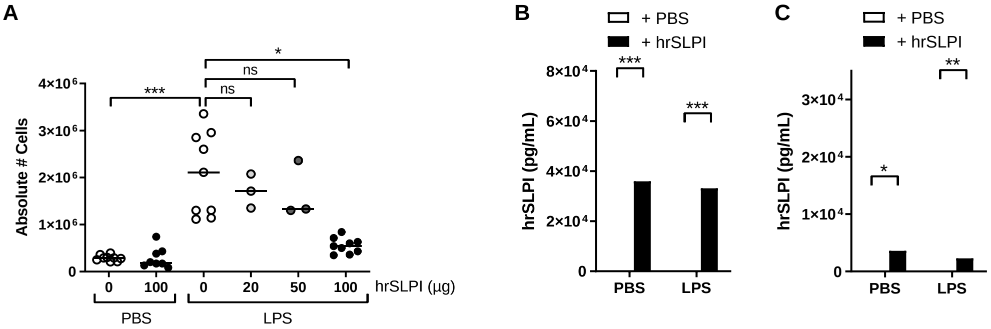

Figure S2. Administration of hrSLPI decreases the LPS-induced expression of numerous inflammatory cytokines. C57bl6 mice were instilled with PBS or 20 µg of LPS intratracheally and injected intraperitoneally with PBS (white dots) or 100 µg hrSLPI (black dots). A cytokine/chemokine array was performed on BAL samples from LPS-instilled animals (**A**; mean +/− sem of 2 technical replicates) and the expression profiles in lung tissue of IL-6, TNFa, IL-1b, IFNg were confirmed by quantitative qRT-PCR (**B**). * ($p < 0.05$) and ** ($p < 0.01$).

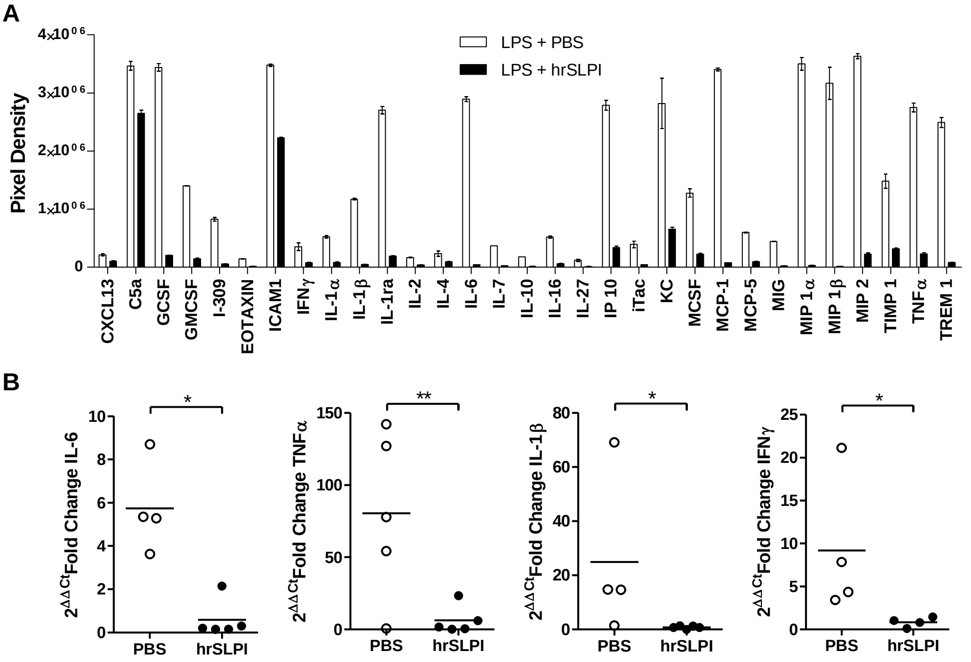

Figure S3. The hrSLPI anti-inflammatory effect is not due to the simple administration of a protein. C57bl6 mice were instilled with 20 µg of LPS intratracheally and injected intraperitoneally with PBS (white dots), 100 µg hrSLPI (black dots) or 100 µg of Ovalbumin (grey dots). After 6 h, the levels of IL-6, KC and MCP-1 were determined by ELISA in the BAL (**A**) and in the serum (**B**) and the total BAL cells were counted (**C**). * (p < 0.05), ** (p < 0.01) or *** (p < 0.001); ns (non-significant) corresponds to p > 0.05.

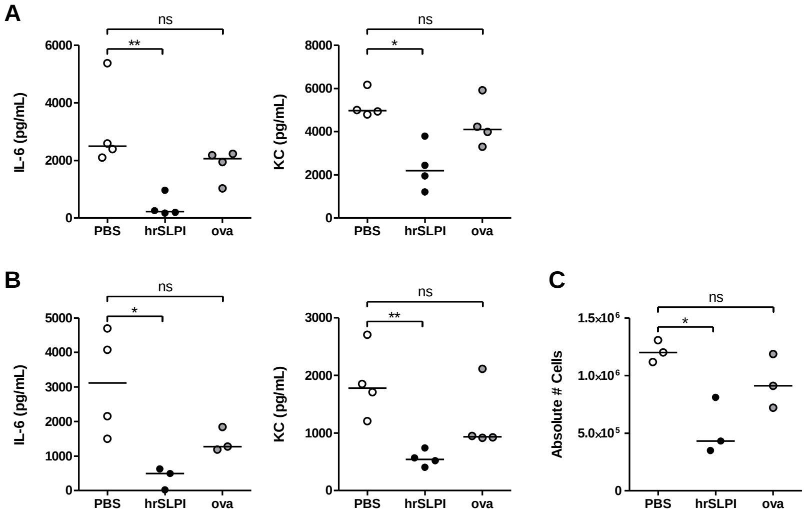

Figure S4. hrSLPI anti-inflammatory properties are not specific of E. coli LPS. C57bl6 mice were injected with 250 µg of *Pseudomonas aeruginosa* LPS (**A**), *Klebsiella pneumoniae* LPS (**B**) or *Staphylococcus aureus* LTA (**C**) intraperitoneally as well as with PBS (white dots) or 100 µg hrSLPI (black dots). After 6 h, the concentration of IL-6 in the lavage and serum was determined by ELISA. * (p < 0.05) and ** (p < 0.01).

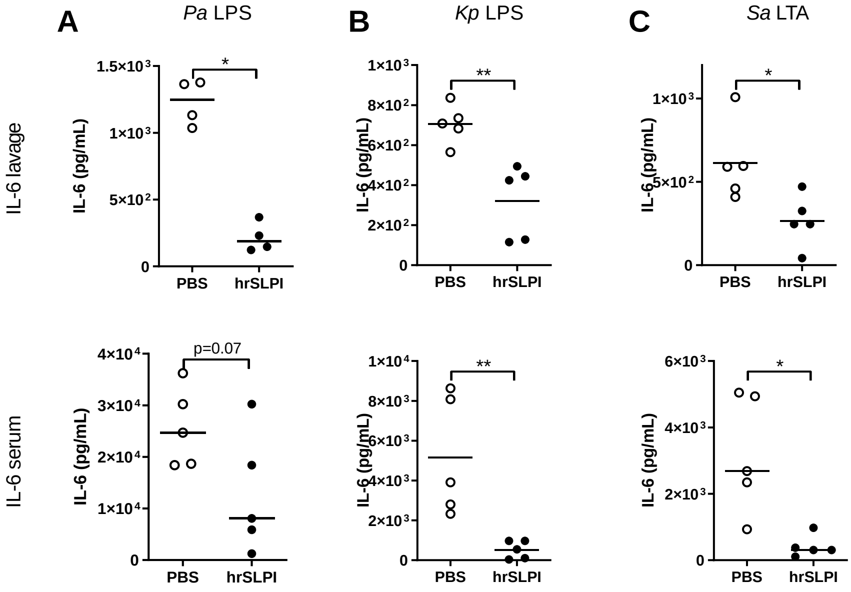

Supplement: Supplementary file 1 [file biomolecules-12-01728-s001.zip › biomolecules-1972221-supplementary.pdf]
